# The thrombelastometry parameter CT_EXTEM_ as an independent risk factor for mortality in bleeding patients

**DOI:** 10.1007/s00068-025-03079-z

**Published:** 2026-02-16

**Authors:** Hagen Bomberg, Klaus Görlinger, Stefan Wagenpfeil, Thomas Volk, Sven Oliver Schneider

**Affiliations:** 1 Department of Anaesthesiology, Intensive Care Medicine and Pain Medicine, Saarland University, University Medical Centre, Forchstrasse 370, Homburg/Saar, Germany; 2 Department of Anaesthesiology, Intensive Care Medicine and Pain Medicine, Balgrist University Hospital, Zurich, Switzerland; 3 Outcomes Research Consortium, Houston, TX USA; 4 Medical Director, Tem Innovations GmbH, Munich, Germany; 5 Institute for Medical Biometry, Epidemiology and Medical Informatics, Saarland University, University Medical Centre, Homburg/Saar, Germany

**Keywords:** Mortality, Risk factor, Bleeding, Rotational thromboelastometry

## Abstract

**Purpose:**

Pathologic thromboelastometric results may indicate a coagulation disorder. During bleeding, the prolongation of CT_EXTEM_ (Clotting Time) measured by rotational thromboelastometry (ROTEM) can detect alterations in the extrinsic pathway. However, the significance of a prolonged CT_EXTEM_ for risk stratification in patients with bleeding remains unclear.

**Methods:**

A total of 2035 consecutive patients were retrospectively examined between 2014 and 2020 from a bleeding database at Saarland University Hospital. The database includes patients tested with ROTEM during bleeding. The study population was split into three groups: Cardiothoracic surgery with cardiopulmonary bypass (CPB, *n* = 753), trauma (*n* = 206) and medical bleeding (*n* = 1076). The impact of CT_EXTEM_ on 30-day mortality was assessed using C-statistic. Threshold values for CT_EXTEM_ reaching a specificity > 90% for 30-day mortality were selected. Adjusted hazard ratios (adjHR [95% confidence interval]) were calculated with multivariable Cox models.

**Results:**

The C-statistic showed that CT_EXTEM_ (C-statistic for groups 1–3: 0.62, threshold ≥ 110 s (CPB); 0.65, threshold ≥ 98 s (trauma); 0.63, threshold ≥ 99 s (medical bleeding)) had a predictive power for 30-day mortality in all groups. The determined threshold value of CT_EXTEM_ reaching a specificity > 90% remained an independent risk predictor for 30-day mortality even after adjustment for confounding factors (CPB: adjHR 2.5 [1.5–4.2], *p* < 0.001; trauma: adjHR 3.9 [1.8–8.7], *p* = 0.001; medical bleeding: adjHR 1.8 [1.4–2.5], *p* < 0.001). The 30-day mortality rate was significantly increased (CPB: CT_EXTEM_ ≥ 110 s, 26% versus 9%, *p* < 0.001; trauma: CT_EXTEM_ ≥ 98 s, 41% versus 11%, *p* < 0.001; medical bleeding: CT_EXTEM_ ≥ 99 s, 41% versus 22%, *p* < 0.001).

**Conclusion:**

Our results indicate that CT_EXTEM_-detected alterations in the extrinsic pathway may be an independent predictor of 30-day mortality in patients with bleeding.

**Supplementary information:**

The online version contains supplementary material available at 10.1007/s00068-025-03079-z.

## Key points

Question: Bleeding can result in coagulation disorders. Rotational thromboelastometry can detect alterations in the extrinsic pathway by measuring clotting time (CT_EXTEM_). The significance of a prolonged CT_EXTEM_ for risk stratification remains unclear.

Findings: 2035 consecutive patients between 2014 and 2020 were retrospectively analysed and threshold value of CT_EXTEM_ remained an independent risk predictor for 30-day mortality.

Meaning: Elevated CT_EXTEM_ could be an independent predictor of 30-day mortality in patients with bleeding.

**Supplementary information:**

The online version contains supplementary material available at 10.1007/s00068-025-03079-z.

## Introduction

Bleeding is associated with rapid loss and consumption of coagulation factors and remains a leading cause of preventable morbidity and mortality in trauma, major, and cardiac surgery with cardiopulmonary bypass. Early, targeted hemostatic therapy is essential, and functional point-of-care methods, such as rotational thromboelastometry (ROTEM), enable the rapid assessment of coagulation deficits [1–3]. 

In EXTEM, coagulation is triggered by tissue factor, initiating clot formation within about 70 s and allowing assessment within 10 min. Key ROTEM parameters include clotting time (CT), clot formation time (CFT), clot firmness (A5, A10, A20, and MCF), and fibrinolytic activity (ML and LI60) [4]. A prolonged clotting time in EXTEM (CT_EXTEM_) in trauma patients was strongly linked to increased mortality. The most common causes of death in trauma patients within the first 24 h are brain injury, followed by haemorrhage [5]. 

Following the algorithm of Weber et al. CT_EXTEM_, A10_EXTEM_, CT_INTEM_, A10_FIBTEM_, and CT_APTEM_ were used for decision-making for coagulation therapy in bleeding patients [3]. Therapeutic strategies for patients with prolonged CT_EXTEM_ commonly include prothrombin complex concentration [3]. 

Our aim was therefore to investigate the relationship between prolonged CT_EXTEM_, 30-day mortality, and the therapeutic addition of prothrombin complex concentrate. Patients were divided into three groups - cardiothoracic surgery with cardiopulmonary bypass, trauma, and medical bleeding- reflecting differences in bleeding pathophysiology. Our primary hypothesis was that there is an independent association between CT_EXTEM_ and 30-day mortality in patients with bleeding.

Second, we hypothesized that prothrombin complex concentrate might improve 30-day survival in patients with prolonged CT_EXTEM_.

## Materials and methods

The study was registered in the German Clinical Trials registration (registration number DRKS00026153) prior to data analysis in September 2021 (principal investigator: PD Dr. med. Sven Oliver Schneider, Saarland University, Department of Anaesthesiology, Intensive Care Medicine and Pain Medicine, Homburg/Saar, Germany). After approval of the Ethical committee (Ärztekammer Saarland; number 93/20), the data were retrospectively collected. The requirement for written informed consent was waived by the Ethical Committee. The data set used in this study is based on the bleeding database of Saarland University Hospital. Data from the bleeding database were previously published about CT_INTEM_ [6]. 

Bleeding episodes were identified and defined according to the treating physician’s clinical judgement. The decision for the treatment with the coagulation factors was made by the treating physician based on the ROTEM-guided bleeding management protocol and the clinical situation. All patients were managed according to the ROTEM-guided bleeding management protocol as described by Weber et al. [3] Clinical practice remained constant throughout the study period (2014–2020), and no subsequent updates to the guideline were implemented during this time. ROTEM testing was systematically performed in all patients with bleeding and suspected coagulation disorders.

### Inclusion/exclusion criteria

All patients tested in the Saarland University Hospital by rotational thromboelastometry between 2014 and 2020 were included. Rotational thromboelastometry is available for all patients with bleeding and/or coagulation disorders in the Saarland University Hospital.

Exclusion criteria for the study population were missing information about CT_EXTEM_ measurement, A10_EXTEM_, CT_INTEM_, A10_FIBTEM_, CT_APTEM,_ and missing information on outcome (Table 2), covariates (Table 1), or implausible data.

**Table 1:** Population characteristics, Comorbidities, and rotational thromboelastometry analysis

	Cardiothoracic surgery with CPB				Trauma				Medical bleeding			
	CT_EXTEM_ < 110s		CT_EXTEM_ ≥ 110s		CT_EXTEM_ < 98s		CT_EXTEM_ ≥ 98s		CT_EXTEM_ < 99s		CT_EXTEM_ ≥ 99s	
	(*n* = 663)		(*n* = 90)		(*n* = 179)		(***n*** = 27)		(*n* = 940)		(*n* = 136)	
*Age (yrs)*	67	(57–74)	67	(55–76)	**61**	**(46–78)**	**77**	**(63–81)#**	64	(56–75)	64	(54–74)
*Male (%)*	476	(72)	64	(71)	112	(63)	17	(63)	555	(59)	87	(64)
*Body mass index (kg/m* ^*2*^ *)*	26	(24–30)	26	(24–29)	26	(23–29)	26	(24–29)	26	(23–29)	26	(23–30)
*Emergency*	106	(16)	10	(11)	136	(76)	23	(85)	**301**	**(32)**	**62**	(46)*
*Re-operation after post-operative bleeding*	**116**	**(17)**	**24**	(27)*	26	(15)	3	(11)	258	(27)	46	(34)
*Comorbidities*												
*Coronary heart disease*	293	(44)	34	(38)	14	(8)	2	(7)	181	(19)	32	(24)
*Cardiac insufficiency (EF < 45%)*	72	(11)	14	(16)	5	(3)	0	(0)	40	(4)	10	(7)
*Chronic obstructive pulmonary disease*	43	(6)	7	(8)	6	(3)	2	(7)	**103**	**(11)**	**25**	**(18)+**
*Insulin-dependent diabetes mellitus*	30	(5)	5	(6)	11	(6)	2	(7)	76	(8)	12	(9)
*Liver disease Child C*	3	(0)	0	(0)	4	(2)	1	(4)	72	(8)	13	(10)
*Coagulation disorders*	**21**	**(3)**	**10**	(11)*	21	(12)	3	(11)	158	(17)	21	(15)
*Anticoagulation prior Rotem analysis*												
*Aspirin 100mg*	284	(43)	35	(39)	18	(10)	0	(0)	136	(14)	24	(18)
*High molecular weight heparin*	40	(6)	6	(7)	**31**	**(17)**	**0**	**(0)#**	202	(21)	32	(24)
*Low molecular weight heparin*	8	(1)	0	(0)	31	(17)	2	(7)	**145**	**(15)**	**8**	**(6)+**
*Other anticoagulation*	**50**	**(8)**	**19**	(21)*	**6**	**(3)**	**5**	**(19)#**	**49**	**(5)**	**17**	**(13)+**
*Rotational thromboelastometry*												
* CT EXTEM*	**74**	**(67–83)**	**142**	(120–184)*	**59**	**(52–68)**	**119**	**(106–151)#**	**61**	**(54–72)**	**132**	**(109–182)+**
* CT INTEM*	***221***	**(195–257)**	***346***	(247–452)*	***161***	**(148–183)**	***230***	**(195–279)#**	***177***	**(155–206)**	***270***	**(209–356)+**
*A10 EXTEM*	***57***	**(50–63)**	***51***	(38–60)*	*53*	(46–59)	*46*	(34–61)	***54***	**(45–63)**	***44***	**(31–58)+**
*A10 FIBTEM*	***13***	**(10–18)**	***13***	(7–17)*	*13*	(9–18)	*10*	(5–17)	***15***	**(10–21)**	***10***	**(4–20)+**
*CT APTEM*	***71***	**(63–82)**	***131***	(116–182)*	***57***	**(49–66)**	***114***	**(101–157)#**	***59***	**(51–70)**	***124***	**(100–172)+**

Continuous variables are expressed as median and interquartile range and were compared with Mann Whitney U test. Categorical variables are presented as numbers (column percentages) and were compared with chi^2^ tests. Threshold values reaching a specificity of > 90% were determined and selected for the analysis. A10: clot formation after 10 min. CT: clotting time in seconds (s). EF: ejection fraction. ROTEM: rotational thromboelastometry. APTEM: extrinsic pathway with Inhibition of fibrinolysis. EXTEM: extrinsic pathway. FIBTEM: fibrin contribution to clot firmness. INTEM: intrinsic pathway without heparin neutralization. The leading department is selected in all 3 groups. Groupe 1: cardiothoracic surgery with CPB includes patients undergoing surgery with cardiopulmonary bypass (CPB). Groupe 2: trauma includes traumatically injured patients, and with 115 patients suffering from multiple injuries that involve multiple organs. Groupe 3: medical bleeding includes all patients with surgery without cardiopulmonary bypass (general surgery (without liver), liver surgery, orthopedic surgery, pediatric, eye, thoracic surgery without CPB, vascular surgery without CPB, gynecology, urology, neurosurgery, ear, nose, throat, and maxillofacial surgery) and 79 patients from internal medicine. Emergency at hospital admission is defined as a surgical intervention necessary within 6 h after hospital admission. Coagulation disorders: known bleeding coagulation disorder, e.g., thrombocytopenia, factor deficiency, von Willebrand disease. Anticoagulation before the Rotem analysis includes anticoagulation in the hospital ward or at home. Other anticoagulants: Argatroban, Phenprocoumon, factor Xa inhibitor, Dabigatran, and P2Y12 inhibitors. * *p* < 0.05 versus CT_EXTEM_ < 110s. # *p* < 0.05 versus CT_EXTEM_ < 98s. + *p* < 0.05 versus CT_EXTEM_ < 99s

### Data source

Rotational thromboelastometry data are recorded during patient care and stored in the hospital’s medical reports and the bleeding database. These include patients with and without surgery. Detailed information about the medical conditions of patients, along with the procedure, was extracted from the hospital database (Tables 1 and 2).

**Table 2:** Outcome related to *CT*_*EXTEM*_* (Clotting Time) *in seconds (s)

Cardiothoracic surgery with CPB										
	Before Rotem analysis					After Rotem analysis				
	CT_EXTEM_ < 110s		CT_EXTEM_ ≥ 110s		*p*-value	CT_EXTEM_ < 110s		CT_EXTEM_ ≥ 110s		*p*-value
	(*n* = 663)		(*n* = 90)			(*n* = 663)		(*n* = 90)		
*Mech. ventilated (h)*	**4**	**(3–6)**	**4**	**(3–9)**	***0.02***	10	(5–19)	8	(1–30)	*0.1*
*Renal replacement therapy (%)*	**53**	**(8)**	**15**	**(17)**	***0.007***	**119**	**(18)**	**30**	**(33)**	***0.001***
*Length of stay (days)*										
*In ICU*	**1**	**(0–1)**	**1**	**(1–1)**	***< 0.001***	2	(1–7)	4	(1–4)	*0.1*
*In hospital*	2	(2–4)	3	(2–6)	*0.3*	10	(7–17)	11	(5–21)	*0.4*
*30-day mortality (%)*	─		─			**59**	**(9)**	**23**	**(26)**	***< 0.001***
*Packed red blood cells (n)*	**0**	**(0–2)**	**1**	**(0–4)**	***0.001***	2	(2–4)	2	(0–5)	*0.3*
*Platelet concentrate (n)*	1	(1–2)	0	(0–2)	*0.1*	2	(2–2)	2	(0–4)	*0.2*
*Fresh frozen plasma (n)*	**0**	**(0–0)**	**0**	**(0–0)**	***0.03***	**0**	**(0–0)**	**0**	**(0–3)**	***0.04***
*Prothrombin complex concentrate (IU)*	0	(0–0)	0	(0–0)	*0.2*	**0**	**(0–0)**	**1500**	**(1500–3000)**	***< 0.001***
*Fibrinogen (g)*	0	(0–0)	0	(0–0)	*0.1*	**0**	**(0–0)**	**0**	**(0–0)**	***0.009***
*Recombinant Factor VIIa (IU)*	0	(0–0)	0	(0–0)	*0.4*	0	(0–0)	0	(0–0)	*0.5*
*Antithrombin III (IU)*	0	(0–0)	0	(0–0)	*0.9*	0	(0–0)	0	(0–0)	*0.7*
*Factor XIII (IU)*	0	(0–0)	0	(0–0)	*0.3*	0	(0–0)	0	(0–0)	*0.7*
*Adverse events*										
*Pneumonia (%)*	22	(3)	4	(4)	*0.5*	104	(16)	15	(17)	*0.8*
*Pulmonary embolism (%)*	5	(1)	3	(3)	*0.1*	5	(1)	1	(1)	*0.5*
*Acute myocardial infarction (%)*	49	(7)	5	(6)	*0.7*	2	(0)	2	(2)	*0.1*
*Embolic apoplexy (%)*	26	(4)	6	(7)	*0.3*	23	(3)	3	(3)	*1*
*Peripheral arterial embolism and thrombosis (%)*	14	(2)	4	(4)	*0.3*	**4**	**(1)**	**4**	**(4)**	***0.009***
*Gastrointestinal ischemia (%)*	**13**	**(2)**	**8**	**(9)**	***0.002***	35	(5)	8	(9)	*0.2*
*Gastrointestinal bleeding (%)*	2	(0)	2	(2)	*0.1*	2	(0)	0	(0)	*1*
***Trauma***										
	***Before Rotem analysis***					***After Rotem analysis***				
	***CT***_***EXTEM***_ ***< 98s***		***CT***_***EXTEM***_ ***≥ 98s***		***p-value***	***CT***_***EXTEM***_ ***< 98s***		***CT***_***EXTEM***_ ***≥ 98s***		***p-value***
	(*n* = 179)		(*n* = 27)			(*n* = 179)		(*n* = 27)		
*Mech. ventilated (h)*	0	(0–2)	0	(0–2)	*1*	1	(0–51)	2	0(−6)	*0.9*
*Renal replacement therapy (%)*	10	(6)	2	(7)	*0.7*	16	(9)	4	(15)	*0.3*
*Length of stay (days)*										
*In ICU*	1	(1–1)	1	(1–1)	*1*	5	(2–15)	7	(1–7)	*0.5*
*In hospital*	2	(1–5)	1	(1–2)	*0.1*	**21**	**(12–34)**	**13**	**(1–22)**	***0.005***
*30-day mortality (%)*	─		─			**19**	**(11)**	**11**	**(41)**	***< 0.001***
*Packed red blood cells (n)*	0	(0–2)	0	(0–1)	*0.4*	2	(0–6)	2	(0–5)	*0.4*
*Platelet concentrate (n)*	0	(0–0)	0	(0–0)	*0.4*	0	(0–1)	0	(0–1)	*0.9*
*Fresh frozen plasma (n)*	0	(0–0)	0	(0–0)	*0.9*	0	(0–0)	0	(0–0)	*0.1*
*Prothrombin complex concentrate (IU)*	0	(0–0)	0	(0–0)	*0.1*	**0**	**(0–0)**	**1000**	**(0–2000)**	***< 0.001***
*Fibrinogen (g)*	0	(0–0)	0	(0–0)	*0.7*	0	(0–0)	0	(0–2)	*0.4*
*Recombinant Factor VIIa (IU)*	0	(0–0)	0	(0–0)	*1*	0	(0–0)	0	(0–0)	*1*
*Antithrombin III (IU)*	0	(0–0)	0	(0–0)	*0.6*	0	(0–0)	0	(0–0)	*0.1*
*Factor XIII (IU)*	0	(0–0)	0	(0–0)	*1*	0	(0–0)	0	(0–0)	*0.4*
*Adverse events*										
*Pneumonia (%)*	21	(12)	0	(0)	*0.1*	13	(7)	2	(7)	*1*
*Pulmonary embolism (%)*	6	(3)	0	(0)	*1*	3	(2)	1	(4)	*0.4*
*Acute myocardial infarction (%)*	1	(1)	0	(0)	*1*	1	(1)	0	(0)	*1*
*Embolic apoplexy (%)*	3	(2)	2	(7)	*0.1*	3	(2)	1	(4)	*0.4*
*Peripheral arterial embolism and thrombosis (%)*	1	(1)	0	(0)	*1*	1	(1)	0	(0)	*1*
*Gastrointestinal ischemia (%)*	1	(1)	0	(0)	*1*	1	(1)	0	(0)	*1*
*Gastrointestinal bleeding (%)*	1	(1)	1	(4)	*0.2*	1	(1)	0	(0)	*1*
***Medical bleeding***										
	***Before Rotem analysis***					***After Rotem analysis***				
	***CT***_***EXTEM***_ ***< 99s***		***CT***_***EXTEM***_ ***≥ 99s***		***p-value***	***CT***_***EXTEM***_ ***< 99s***		***CT***_***EXTEM***_ ***≥ 99s***		***p-value***
	(*n* = 940)		(*n* = 136)			(*n* = 940)		(*n* = 136)		
*Mech. ventilated (h)*	**0**	**(0–5)**	**1**	**(0–7)**	***0.003***	2	(0–22)	3	(0–26)	*0.2*
*Renal replacement therapy (%)*	**136**	**(14)**	**30**	**(22)**	***0.03***	**188**	**(20)**	**49**	**(36)**	***< 0.001***
*Length of stay (days)*										
*In ICU*	**1**	**(1–2)**	**1**	**(1–2)**	***0.02***	**4**	**(1–12)**	**7**	**(2–14)**	***0.03***
*In hospital*	2	(2–9)	2	(1–12)	*1*	**17**	**(8–30)**	**13**	**(4–30)**	***0.01***
*30-day mortality (%)*	─		─			**203**	**(22)**	**56**	**(41)**	***< 0.001***
*Packed red blood cells (n)*	0	(0–4)	1	(0–4)	*0.2*	2	(0–5)	2	(0–5)	*0.5*
*Platelet concentrate (n)*	0	(0–0)	0	(0–0)	*0.5*	**0**	**(0–1)**	**0**	**(0–2)**	***0.04***
*Fresh frozen plasma (n)*	0	(0–0)	0	(0–0)	*0.2*	**0**	**(0–0)**	**0**	**(0–2000)**	***0.005***
*Prothrombin complex concentrate (IU)*	**0**	**(0–0)**	**0**	**(0–875)**	***0.004***	**0**	**(0–0)**	**0**	**(0–2)**	***< 0.001***
*Fibrinogen (g)*	0	(0–0)	0	(0–0)	*0.4*	**0**	**(0–0)**	**0**	**(0–0)**	***< 0.001***
*Recombinant Factor VIIa (IU)*	0	(0–0)	0	(0–0)	*0.5*	0	(0–0)	0	(0–0)	*0.6*
*Antithrombin III (IU)*	0	(0–0)	0	(0–0)	*0.4*	0	(0–0)	0	(0–0)	*1*
*Factor XIII (IU)*	0	(0–0)	0	(0–0)	*0.3*	0	(0–0)	0	(0–0)	*0.7*
*Adverse events*										
*Pneumonia (%)*	**113**	**(12)**	**27**	**(20)**	***0.01***	63	(7)	9	(7)	*1*
*Pulmonary embolism (%)*	22	(2)	4	(3)	*0.6*	7	(1)	2	(1)	*0.3*
*Acute myocardial infarction (%)*	27	(3)	2	(1)	*0.6*	6	(1)	1	(1)	*1*
*Embolic apoplexy (%)*	24	(3)	3	(2)	*1*	3	(0)	1	(1)	*0.4*
*Peripheral arterial embolism and thrombosis (%)*	**40**	**(4)**	**12**	**(9)**	***0.03***	2	(0)	2	(1)	*0.1*
*Gastrointestinal ischemia (%)*	44	(5)	12	(9)	*0.1*	8	(1)	1	(1)	*1*
*Gastrointestinal bleeding (%)*	101	(11)	16	(12)	*0.8*	11	(1)	3	(2)	*0.4*

The outcome was split into the time before and after the Rotem analysis. Continuous variables are expressed as median and interquartile range and were compared with Mann Whitney U test. Categorical variables are presented as numbers (column percentages) and were compared with chi tests. Threshold values reaching a specificity of >90% were determined and selected for the analysis. ICU (Intensive care unit). Rotem (Rotational thromboelastometry). Packed red blood cells (450mL), Platelet concentrate (270mL), and Fresh frozen plasma (300mL). CT: clotting time in seconds (s). EXTEM: extrinsic pathway. The leading department is selected in all 3 groups. Groupe 1: Cardiothoracic surgery with CPB includes patients undergoing surgery with cardiopulmonary bypass (CPB). Groupe 2: Trauma includes traumatically injured patients, with 115 patients suffering from multiple injuries that involve multiple organs. Groupe 3: Medical bleeding includes all patients with surgery without cardiopulmonary bypass (general surgery (without liver), liver surgery, orthopedic surgery, pediatric, eye, thoracic surgery without CPB, vascular surgery without CPB, gynecology, urology, neurosurgery, ear, nose, throat, and maxillofacial surgery) and 79 patients from internal medicine

### Rotational thromboelastometry analyses

The ROTEM (ROTEM delta^®^, Tem Innovations, Munich, Germany) analyses in this study include measurements of tissue factor–activated EXTEM (extrinsic pathway activation without heparin neutralization), FIBTEM (fibrin contribution to clot firmness), and APTEM (extrinsic pathway with inhibition of fibrinolysis). All assays estimate clotting time (CT) and clot formation after 10 min (A10). The reference range for CTEXTEM on our ROTEM thromboelastometry device is 38–82 s. Each patient was considered only once. If more than one ROTEM analysis was done in one patient, only the first analysis was considered.

### Proof of plausibility

Data integrity was evaluated according to specific rules to delete erroneously entered data and cases with missing information (proof of plausibility, Appendix 1).

### Missing data handling/sensitivity analyses

Patients with missing data were excluded from the analysis. Several sensitivity analyses were performed.

### Definitions of the groups

- Groupe 1: Cardiothoracic surgery with CPB includes patients undergoing surgery with cardiopulmonary bypass (CPB).
- Groupe 2: Trauma includes traumatically injured patients, and with 115 patients suffering from multiple injuries that involve multiple organs.
- Groupe 3: Medical bleeding includes all patients with surgery without cardiopulmonary bypass (general surgery (without liver), liver surgery, orthopedic surgery, pediatric, eye, thoracic surgery without CPB, vascular surgery without CPB, gynecology, urology, neurosurgery, ear, nose, throat, and maxillofacial surgery) and 79 patients from internal medicine.
- Subgroup 1: Patients without anticoagulation (without high molecular weight heparin, low molecular weight heparin, Argatroban, Phenprocoumon, Factor Xa inhibitor, Dabigatran, and P2Y12 inhibitors). Aspirin 100 mg was not considered anticoagulant.
- Subgroup 2: Patients treated with packed red blood cells ≥ 6.

### Definition of the outcomes

The primary outcome was overall survival with a follow-up period of 30 days.

The secondary outcomes were: Severe adverse events were defined by the occurrence of pneumonia, pulmonary embolism, acute myocardial infarction, embolic apoplexy, peripheral arterial embolism, thrombosis, and gastrointestinal bleeding. Gastrointestinal ischemia was defined as occlusive or non-occlusive mesenteric ischemia, with diagnosis confirmed by laparotomy, computed tomography, or mesenteric angiography.

### Data analysis

Receiver operating characteristic (ROC) curves were constructed (C-statistic) to evaluate the predictive power of CT_EXTEM_ for the occurrence of 30-day mortality. The Youden Index was used to calculate the optimal threshold for CT_EXTEM_ in predicting 30-day mortality. Threshold values achieving a specificity of > 90% were determined with their corresponding specificities and selected for analysis. Continuous variables are expressed as median and interquartile range and were compared with Mann Whitney U test. Categorical variables are presented as absolute and relative frequencies and were compared with chi^2^ tests.

The number needed to screen was calculated to measure how many patients must be screened with values CT_EXTEM_ ≥ 110 s, CT_EXTEM_ ≥ 98 s, and CT_EXTEM_ ≥ 99 s to avoid one death. The positive predictive value was defined as the number of true positives/(number of true positives + number of false positives). The negative predictive value was defined as the number of true negatives/(number of true negatives + number of false negatives).

Survival rates were estimated using the Kaplan-Meier method and compared using the log-rank test. Cox proportional hazards models were used to adjust for confounding factors, including all variables from Table 1 with *p* < 0.05. Pairwise dependent variable constellations with Pearson or Spearman correlation coefficients exceeding + 0.4 or less than − 0.4 were a priori specified as interaction terms in multivariable analyses to account for the issue of multicollinearity. CT_EXTEM_ was analysed a threshold value and as a continuous variable (per 10-second increase). Sensitivity analyses were performed, including confounding factors listed in Tables 1 and 2.

We developed a propensity score for each patient based on the potential confounders listed in Tables 1 and 2. Patients given prothrombin complex concentrate were matched to patients with the closest propensity score who were not given prothrombin complex concentrate, keeping the maximum difference in propensity score less than 0.05. The matching algorithm was nearest neighbour matching without replacement. Two-sided p-values > 0.1 were considered balanced. Data analysis was performed using SPSS Statistics 29™ (IBM, Ehningen, Germany). Two-sided p-values < 0.05 were considered statistically significant.

## Results

During the study period, 2656 patients had documented index rotational thromboelastometry (ROTEM) analyses. Among these, 375 patients had no information about CT_EXTEM_ analysis, 74 patients had missing information on outcome, covariates, or implausible data, and 172 patients had missing information about A10_EXTEM_, CT_INTEM_, A10_FIBTEM_, and CT_APTEM_. The final study population included 2035 patients and was divided into three groups: Cardiothoracic surgery with cardiopulmonary bypass (CPB, *n* = 753), trauma (*n* = 206), and medical bleeding (*n* = 1076).

**Fig. 1:**
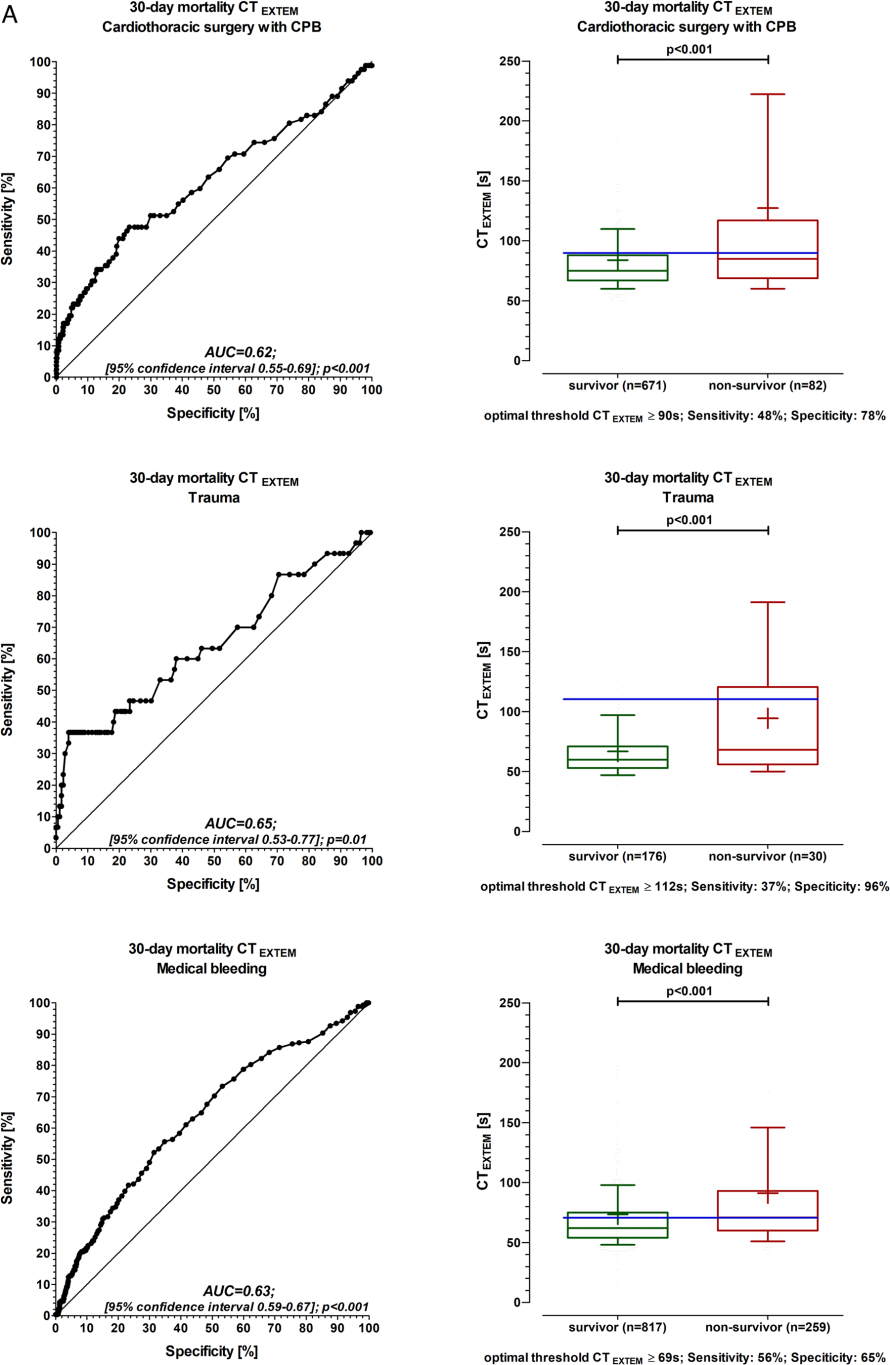

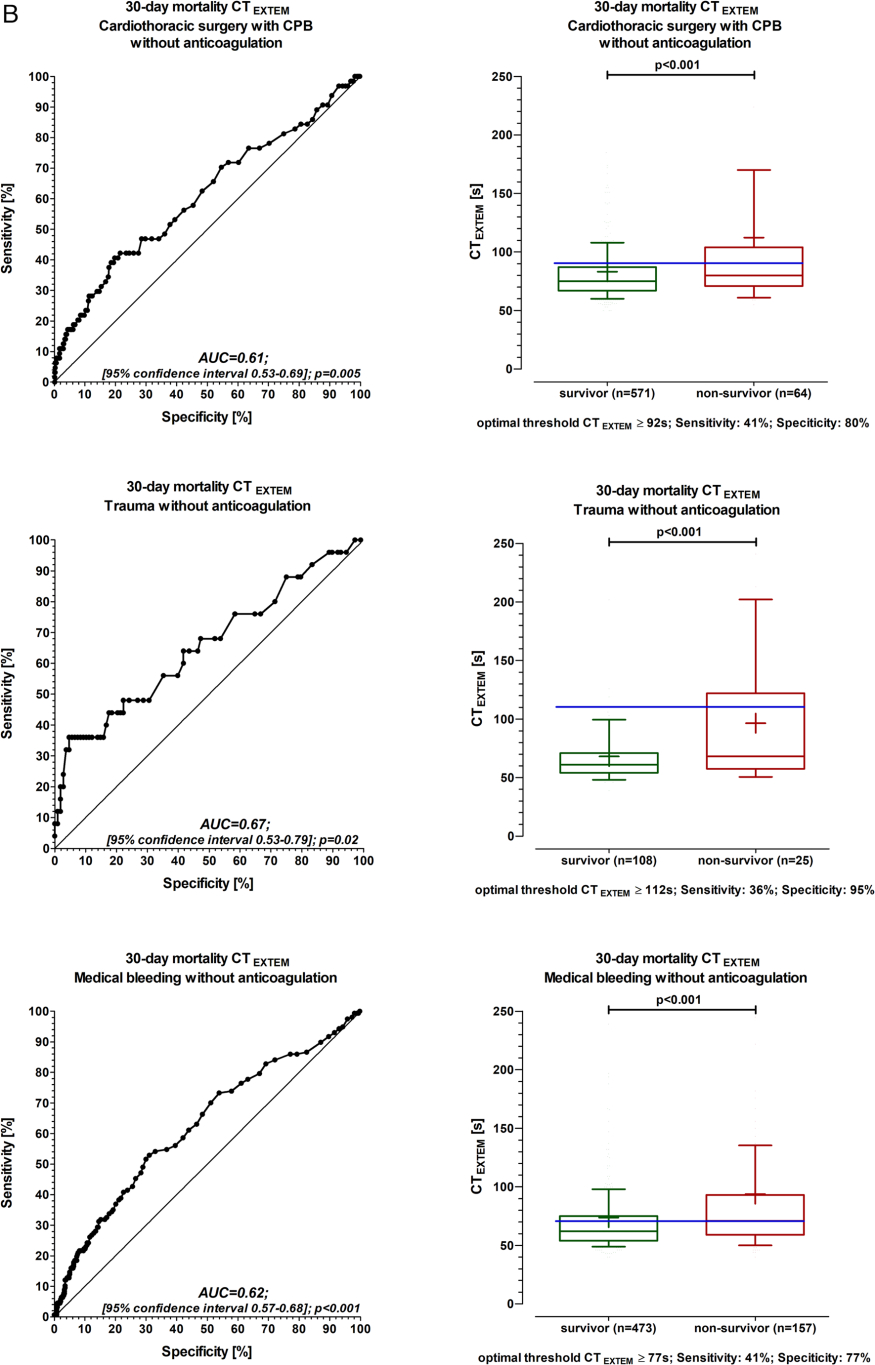
CT_EXTEM_ (Clotting Time) and ROC analysis for mortality in all patients. Figure 1B: CT_EXTEM_ (Clotting Time) and ROC analysis for mortality in patients without anticoagulation Box and whisker plot with the 10th and 90th percentiles. Solid dash: median values of CT_EXTEM_. The plus symbol: mean values of CT_EXTEM_. Blue solid dashed line: optimal threshold. Receiver operating characteristic curves (ROC) were constructed (C-statistic) to evaluate the predictive power of CT_EXTEM_ for 30-day mortality. AUC (area-under-the-curve). The Youden Index was used to calculate the optimal threshold for CT_EXTEM_ in the prediction of 30 days. Data were collected in real time by the attending physician or nurse in parallel to the patient’s treatment. Each patient was considered only once. If more than one Rotem (Rotational thromboelastometry) analysis was done for one patient, only the first Rotem analysis was considered. The leading department is selected in all 3 groups. Groupe 1: Cardiothoracic surgery with CPB includes patients undergoing surgery with cardiopulmonary bypass (CPB). Groupe 2: Trauma includes traumatically injured patients, and with 115 patients suffering from multiple injuries that involve multiple organs. Groupe 3: Medical bleeding includes all patients with surgery without cardiopulmonary bypass (general surgery (without liver), liver surgery, orthopaedic surgery, paediatric, eye, thoracic surgery without CPB, vascular surgery without CPB, gynaecology, urology, neurosurgery, ear, nose, throat, and maxillofacial surgery) and 79 patients from internal medicine

The C-statistic showed that CT_EXTEM_ predicted 30-day mortality in all three groups (Fig. 1A). The number needed to screen (NNS), the positive predictive value (PPV), and the negative predictive value (NPV) were calculated for 30-day mortality using the threshold value of CT_EXTEM_ reaching a specificity of > 90% (CPB: CT_EXTEM_ ≥ 110 s (NNS: 6, PPV: 0.26, NPV: 0.91; trauma: CT_EXTEM_ ≥ 98 s (NNS: 3, PPV: 0.48, NPV: 0.88; medical bleeding: CT_EXTEM_ ≥ 99 s (NNS: 5, PPV: 0.41, NPV: 0.78).

Patients with a threshold value of CT_EXTEM_ reaching a specificity > 90% were quite comparable in their population characteristics to patients with lower CT_EXTEM_ values (Table 1). However, diagnostic rotational thromboelastometry shows impaired blood clotting in all groups (Table 1).

The outcome was split into the time before and after the ROTEM analysis. Patients with increased CT_EXTEM_ had more renal replacement therapy and required more coagulation factors (Table 2).

**Fig. 2:**
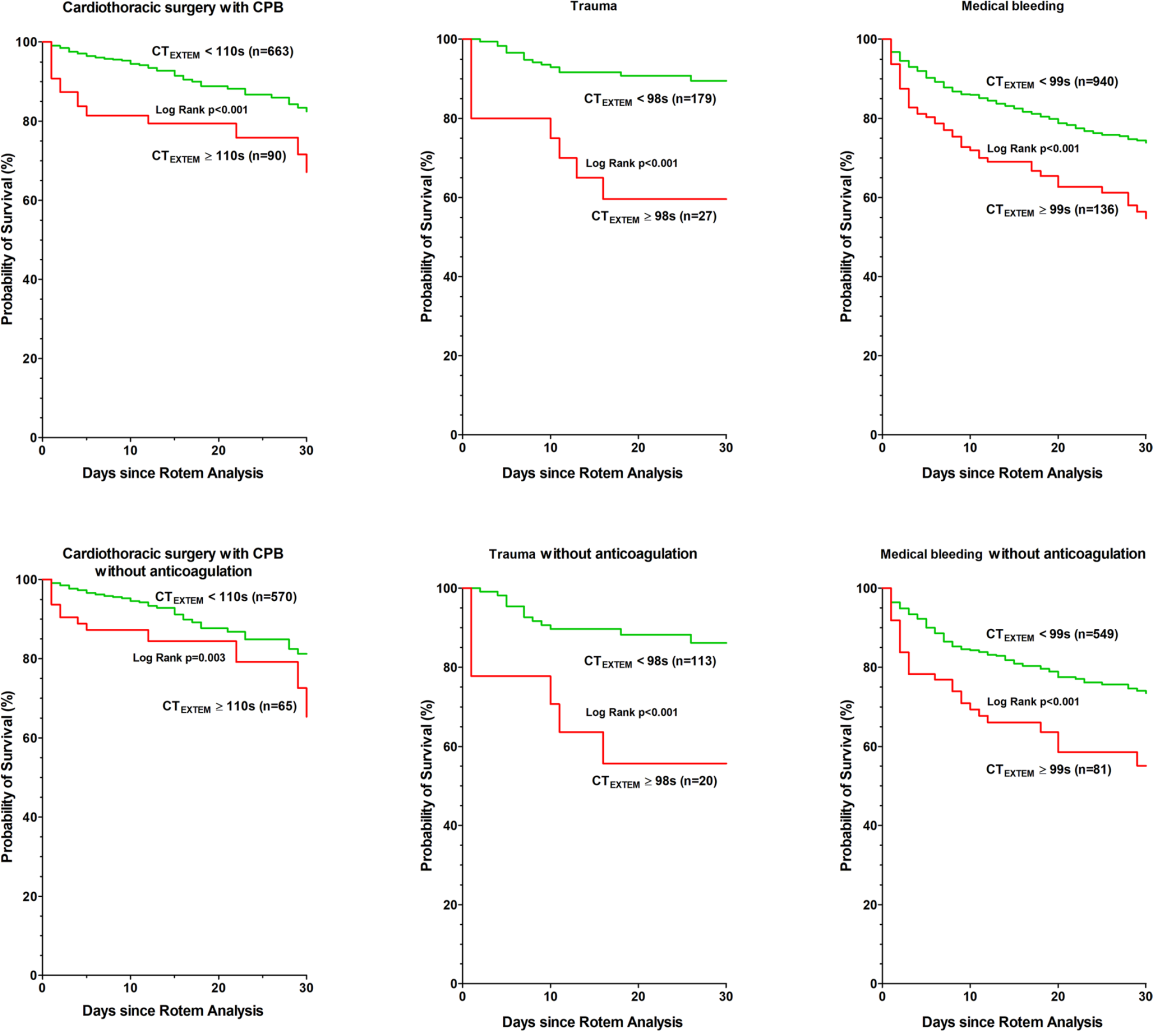
Kaplan-Meier survival plots for elevated CT_EXTEM_ versus lower CT_EXTEM_ in all patients and in patients without anticoagulation (except aspirin 100 mg) during 30-day follow-up. Rotem (Rotational thromboelastometry). The leading department is selected in all 3 groups. Groupe 1: Cardiothoracic surgery with CPB includes patients undergoing surgery with cardiopulmonary bypass (CPB). Groupe 2: Trauma includes traumatically injured patients, and with 115 patients suffering from multiple injuries that involve multiple organs. Groupe 3: Medical bleeding includes all patients with surgery without cardiopulmonary bypass (general surgery (without liver), liver surgery, orthopaedic surgery, paediatric, eye, thoracic surgery without CPB, vascular surgery without CPB, gynaecology, urology, neurosurgery, ear, nose, throat, and maxillofacial surgery) and 79 patients from internal medicine

Patients with elevated CT_EXTEM_ had an increased 30-day mortality compared to patients with lower CT_EXTEM_ (CPB: CT_EXTEM_ ≥ 110 s, 26% versus 9%, *p* < 0.001; trauma: CT_EXTEM_ ≥ 98 s, 41% versus 11%, *p* < 0.001; medical bleeding: CT_EXTEM_ ≥ 99 s, 41% versus 22%, *p* < 0.001; Table 2; Fig. 2). Even after adjustment, elevated CT_EXTEM_ remained an independent risk factor for 30-day mortality. This remained true in sensitivity analyses for the CPB and the medical bleeding group (Table 3). In the trauma group with a smaller sample size, the results were not significant (Table 3).

**Table 3:** Cox proportional hazards models: CT EXTEM threshold a risk factor for 30-day mortality

	Cardiothoracic surgery with CPB		Trauma		Medical bleeding		All patiens	
	(*n* = 753)		(*n* = 206)		(*n* = 1076)		(*n* = 2035)	
	CT_EXTEM_≥ 110s		CT_EXTEM_≥ 98s		CT_EXTEM_ ≥ 99s		CT_EXTEM_	
***Model***	***HR (95% CI)***	***p-value***	***HR (95% CI)***	***p-value***	***HR (95% CI)***	***p-value***	***HR (95% CI)***	***p-value***
***crude in all patients***	2.9 (1.8–4.7)	***< 0.001***	4.9 (2.3–10.4)	***< 0.001***	2.1 (1.6–2.9)	***< 0.001***	1.03 (1.02–1.03)	***< 0.001***
***Primary model***								
* adjusted all population characteristics*,* comorbidities with p* < 0.05	2.5 (1.5–4.2)	***< 0.001***	3.9 (1.8–8.7)	***0.001***	1.8 (1.4–2.5)	***< 0.001***	1.03 (1.02–1.03)	***< 0.001***
***Sensitivity Analyses***								
*adjusted all population characteristics*,* comorbidities*	2.9 (1.7–4.8)	***< 0.001***	2.6 (1.1–6.2)	***0.03***	1.9 (1.4–2.6)	***< 0.001***	1.03 (1.02–1.04)*	***< 0.001***
*adjusted all population characteristics*,* comorbidities*,* CT INTEM and A10 FIBTEM*	2.7 (1.6–4.6)	***< 0.001***	1.1 (0.3–3.4)	*0.9*	1.6 (1.2–2.3)	***0.003***	1.04 (1.02–1.05)*	***< 0.001***
*adjusted all population characteristics*,* comorbidities*,* CT INTEM and A10 FIBTEM*,* all variables before Rotem analysis from* Table 2	2.3 (1.2–4.3)	***0.01***	2.0 (0.5–7.8)	*0.3*	1.5 (1.1–2.1)	***0.03***	1.03 (1.01–1.05)*	***0.003***
*adjusted all population characteristics*,* comorbidities*,* CT *INTEM *and A10 *FIBTEM,* all variables before Rotem analysis from* Table 2, *year of Rotem analysis*	2.2 (1.2–4.2)	***0.02***	2.4 (0.6–10.2)	*0.2*	1.5 (1.1–2.1)	***0.03***	1.03 (1.01–1.05)*	***< 0.001***

Primary model and sensitivity analyses. Data are expressed as HR (hazard ratio) with a 95% confidence interval (CI). Hazards ratio of CT_EXTEM_ (Clotting Time) in seconds (s) adjusted for confounders. Cox proportional hazard models were used to adjust for confounding factors. Rotem (Rotational thromboelastometry). Threshold values reaching a specificity of >90% were determined and selected for the analysis. In all patients. CT_EXTEM_ was analysed as a threshold value and as a continuous variable (per 10-second increase). * Adjusted for medical departments Rotem: Rotational thromboelastometry. APTEM: extrinsic pathway with inhibition of fibrinolysis. EXTEM: extrinsic pathway. FIBTEM: fibrin contribution to clot firmness. INTEM: intrinsic pathway without heparin neutralization. The leading department is selected in all 3 groups. Groupe 1: Cardiothoracic surgery with CPB includes patients undergoing surgery with cardiopulmonary bypass (CPB). Groupe 2: Trauma includes traumatically injured patients, with 115 patients suffering from multiple injuries that involve multiple organs. Groupe 3: Medical bleeding includes all patients with surgery without cardiopulmonary bypass (general surgery (without liver), liver surgery, orthopedic surgery, pediatric, eye, thoracic surgery without CPB, vascular surgery without CPB, gynecology, urology, neurosurgery, ear, nose, throat, and maxillofacial surgery) and 79 patients from internal medicine

In several subgroup analyses C-statistic showed similar results compared to the final study population. In patients without anticoagulation, the results were similar (Figs. 1B and 2). In patients treated with packed red blood cells ≥ 6, the results were similar as well (threshold value of CT_EXTEM_ reaching a specificity of > 90% CT_EXTEM_ ≥ 103 s, C-statistic 0.62, sensitivity 30%, and specificity 90%). Also, in a larger population of 2207 patients, including 172 patients with missing information, the results were similar (threshold value of CT_EXTEM_ reaching a specificity of > 90%, CT_EXTEM_ ≥ 109 s, sensitivity 26%, and specificity 90%).

We compared survivors versus non-survivors in patients suffering from elevated CT_EXTEM_. Interestingly, adverse events were quite comparable; however, rotational thromboelastometry shows in all non-survivors an impaired blood clotting (Appendix 2).

**Fig. 3:**
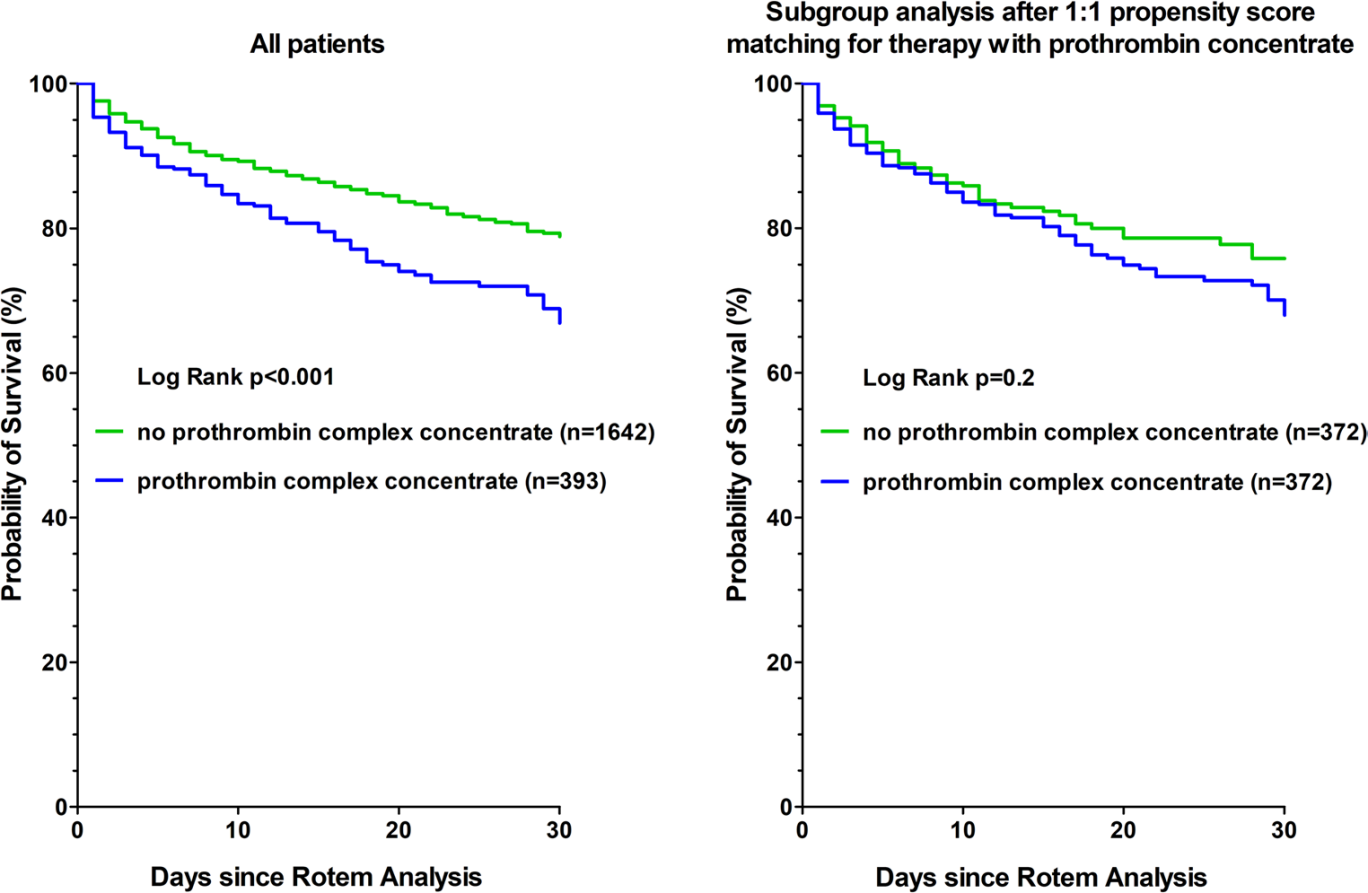
Kaplan-Meier survival plots for therapy with prothrombin complex concentrate versus no therapy with prothrombin complex concentrate during 30-day follow-up for all patients and in subgroup analysis after 1:1 propensity score matching for therapy with prothrombin concentrate. CT_EXTEM_ (Clotting Time). Rotem (Rotational thromboelastometry)

One therapeutic approach for patients with elevated CT_EXTEM_ is the administration of prothrombin complex concentrate. Propensity matching successfully paired 372 without and 372 with treatment of prothrombin complex concentrate. The matched groups were balanced on covariables (smallest p-value > 0.2). In this population, prothrombin complex concentrate is not associated with an increased 30-day mortality rate, in contrast to all patients (Fig. 3).

## Discussion

Given the distinct underlying pathophysiology, patients were categorized into three main groups – cardiothoracic surgery with cardiopulmonary bypass, trauma and medical bleeding – and further stratified into two clinically relevant subgroups (pre-existing anticoagulation and massive transfusion defined as > 6 packed red blood cells). Group-specific thresholds were applied to the specificity of more than 90% (CT_EXTEM_ over ≥ 110 s for cardiothoracic surgery, ≥ 98 s for trauma, and ≥ 99 s for medical bleeding). Despite this heterogeneity, prolonged CT_EXTEM_ above these thresholds was consistently associated with higher transfusion requirements as a marker of bleeding severity and with increased mortality across cohorts.

Mortality in our cardiothoracic surgery and medical bleeding cohorts was 26% and 41%, respectively. Overall 30-day mortality in our trauma cohort was 41%, which is higher than reported, reflecting our small sample size [7, 8]. 

Callcut et al. found that trauma patients most commonly died from traumatic brain injury, followed by haemorrhage [5]. Deaths related to bleeding peaked within the first 24 h [5, 7]. 

In this study, bleeding was defined at the discretion of the treating physician. No universal definition of major bleeding exists [9]. The International Society on Thrombosis and Hemostasis provides a standardized definition for non-surgical patients and proposes a less objective definition for surgical patients [10, 11]. Management principles are largely similar; however, the underlying pathophysiology varies.

Bleeding in trauma as well as surgical patients results from a complex interaction of different pathophysiologic mechanisms resulting in an acquired platelet dysfunction, consumption of coagulation factors, activation of fibrinolytic pathways, and an increased inflammatory response [12, 13]. 

Ongoing haemorrhage, haemorrhagic shock, trauma-induced coagulopathy, and inadequate resuscitation cause early mortality. Risk factors associated with complications and death after significant bleeding include advanced age, coagulation disorders, emergency admission, acute kidney and lung injury, and gastrointestinal ischemia [8, 14–18]. 

Even after adjusting for potential confounders (Table 3), prolonged CT_EXTEM_ over ≥ 110 s for cardiothoracic surgery, ≥ 98 s for trauma, and ≥ 99 s for medical bleeding further remained an independent risk factor for 30-day mortality. In trauma, the association is less consistent, likely due to a small sample size, and the results must be interpreted with caution.

We hypothesize that the primary cause of death with an increased CT_EXTEM_ may be related to haemostatic dysfunction within the extrinsic pathway.

Prolongation of CT_EXTEM_ reflects impaired initiation of coagulation in a tissue factor-activated system and is primarily influenced by factor VII-dependent thrombin generation and the availability of coagulation substrates. Because EXTEM initiates coagulation via tissue factor, deficiencies or functional impairment of factor VII — whether inherited or acquired (e.g., liver dysfunction, vitamin K deficiency, anticoagulation, or disseminated intravascular coagulation) — can lead to delayed clot initiation. In addition, reduced fibrinogen concentration limits effective thrombin-mediated fibrin formation and may further prolong CT_EXTEM_, highlighting the substrate dependence of early clot formation.

Beyond factor deficiencies, global physiology disturbances such as haemodilution, acidosis, and hypothermia impair enzymatic activity and thrombin generation, contributing to prolonged CT_EXTEM_ [19, 20]. Therapeutic administration of blood products, like fibrinogen and thrombocyte concentrate, can lead to significant improvement of CT_EXTEM_ [21–25]. Hence, the sequence of haemostatic therapy is important. Fibrinogen, often the first factor to drop, should be replaced early, typically with tranexamic acid [26–28]. If CT_EXTEM_ remains prolonged, prothrombin complex concentrate should be given [3]. However, some institutions use fresh frozen plasma instead of prothrombin complex concentrate, depending on local transfusion protocols [29]. Some studies have reported that prothrombin complex concentrate is more effective than fresh frozen plasma in this setting; however, most of this evidence comes from cardiac surgery populations [30–32]. 

At our hospital, we followed recommendations by Weber et al. for goal-directed rotational thrombelastography-based coagulation management during major bleeding [3]. According to these recommendations, prothrombin complex concentrate was administered at a CT_EXTEM_ >80 s. However, newer recommendations from 2024 substitute prothrombin complex concentrate only when CT_EXTEM_ exceeds 100 s, which is consistent with the patterns observed in our results [13, 33]. 

Furthermore, the administration of prothrombin complex concentrate was associated with a higher incidence of thromboembolic events [34], probably due to an increase in thrombin generation [25]. This coincides with our results, showing that peripheral arterial embolism and thrombosis in our group of patients undergoing cardiothoracic surgery with cardiopulmonary bypass, with CT_EXTEM_ >110 s, were more frequent. Renal replacement therapy and coagulation therapy, especially requiring more fresh frozen plasma, prothrombin complex, and fibrinogen, remained higher in the group of cardiothoracic surgery and medical bleeding. The study by Cappabianca et al. showed an increased risk for acute kidney injury with the use of prothrombin complex concentrate [35]. However, the incidence of acute kidney injury was significantly lower in the prothrombin complex concentrate compared with the fresh frozen plasma group in the randomized multicentre FARES-II trial [30]. This is in agreement with the studies published by Görlinger et al. and Weber et al. more than 10 years ago [2, 36]. In our study, after propensity score matching, prothrombin complex concentrate is not associated with an increased 30-day mortality rate in bleeding patients. In contrast, in 13 healthy volunteers, conflicting results were published, showing no effect of prothrombin complex concentrate in CT_EXTEM_ [37]. Therefore, applying prothrombin complex concentrate is to be avoided in patients without bleeding with prolonged CT_EXTEM_.

The most important limitation is that our analysis is based on a single-centre study population. However, in several sensitivity analyses, we were able to confirm the consistency of our results. Another limiting factor is the lack of detailed information on the sequence of haemostatic therapy – specifically, whether prothrombin complex concentrate was administered as the initial intervention or preceded by agents such as fibrinogen, tranexamic acid, or factor XIII. This is relevant, as previous studies have demonstrated that fibrinogen administration alone can shorten CT_EXTEM_ values [21, 22]. 

Another key limitation of our study is the small size of the trauma subgroup, which consisted primarily of severely injured polytrauma patients with a high mortality rate (41%). Consequently, sensitivity analyses within this group were heavily adjusted and must be interpreted with caution. These factors may limit the generalizability of our findings to broader trauma populations. Accordingly, another limitation of our study is that CT_EXTEM_ results were analysed in isolation and not together with A5_EXTEM_ and A5_FIBTEM_ [23, 24, 38]. Therefore, a larger multicentre study population is needed to confirm our results.

In conclusion, in our study, CT_EXTEM_ is an independent risk factor for 30-day mortality in bleeding patients. After multivariate analysis, bleeding patients with prothrombin complex concentrate do not seem to be associated with increased 30-day mortality. However, fibrinogen levels should be corrected before prothrombin complex concentrate administration, particularly in this high-risk subgroup with evidence of impaired clotting via the extrinsic pathway.

## Supplementary information

Below is the link to the electronic supplementary material.

ESM 1 (DOCX 20.1 KB)

ESM 2 (DOCX 28.2 KB)

## Data Availability

Data will be made available on reasonable request.
