# Supplementary material for: The thrombelastometry parameter CTEXTEM as an independent risk factor for mortality in bleeding patients
Source: Eur J Trauma Emerg Surg. 2026 Feb 16;52(1):47. doi: 10.1007/s00068-025-03079-z (PMC12909406; doi:10.1007/s00068-025-03079-z)
Supplement: Supplementary file 1 — (DOCX 20.1 KB) [file 68_2025_3079_MOESM1_ESM.docx]

**Appendix 1: Proof of plausibility**

| **Items in the registry** | - **Indications of implausible data** |
| --- | --- |
| Day of rotational thromboelastometry analysis | - Implausible if rotational thromboelastometry analysis is performed before admission or after discharge from hospital |
| Age | - Implausible if rotational thromboelastometry analysis takes place before date of birth |
| Body mass index | - Implausible if body height and body weight are not consistent (e.g. height: 180 cm, weight: 10 kg) |
| Emergency | - Implausible if the case was not noted as an emergency in the medical records |
| Department | - Implausible if the department cannot be found in the medical records |
| Re-operation after bleeding | - Implausible if the case was not noted as a re-operation after bleeding in the medical records |
| Comorbidities | - Implausible if the comorbidities cannot be found in the medical records |
| Anticoagulation | - Implausible if the anticoagulation cannot be found in the medical records |
| 30-day mortality | - Implausible if the patient was discharged alive after 30 days or is still living in hospital after 30 days |
| Renal replacement therapy | - Implausible if the renal replacement therapy was not found in the medical records |
| Adverse events | - Implausible if the adverse events cannot be found in the medical records |
| All variables in Table 2, except renal replacement therapy, mortality and adverse events | - Implausible if the total number recorded in the medical records does not match the sum of the time points before the rotational thromboelastometry analysis and after the rotational thromboelastometry analysis (e.g. the total number of administrated packed red blood cells during the hospital stay = administrated packed red blood cells before the rotational thromboelastometry analysis **plus** administrated packed red blood cells after the rotational thromboelastometry analysis) |
