# Supplementary material for: The thrombelastometry parameter CTEXTEM as an independent risk factor for mortality in bleeding patients
Source: Eur J Trauma Emerg Surg. 2026 Feb 16;52(1):47. doi: 10.1007/s00068-025-03079-z (PMC12909406; doi:10.1007/s00068-025-03079-z)
Supplement: Supplementary file 2 — (DOCX 28.2 KB) [file 68_2025_3079_MOESM2_ESM.docx]

**Appendix 2**

| ***Appendix 2*** | | | | | | | | | | | | |
| --- | --- | --- | --- | --- | --- | --- | --- | --- | --- | --- | --- | --- |
| ***30-day mortality*** | | | | | | | | | | | | |
|  | ***Cardiothoracic surgery with CPB*** | | | | ***Trauma*** | | | | ***Medical bleeding*** | | | |
|  | ***CT_EXTEM_ ≥ 110s*** | | | | ***CT_EXTEM_ ≥ 98s*** | | | | ***CT_EXTEM_ ≥ 99s*** | | | |
|  | ***survivor*** | | ***non-survivor*** | | ***survivor*** | | ***non-survivor*** | | ***survivor*** | | ***non-survivor*** | |
|  | ***(n=67)*** | | ***(n=23)*** | | ***(n=16)*** | | ***(n=11)*** | | ***(n=80)*** | | ***(n=56)*** | |
|  |  |  |  |  |  |  |  |  |  |  |  |  |
|  |  |  |  |  |  |  |  |  |  |  |  |  |
| *Mech. ventilated (h)* | 6 | (1-27) | 21 | (1-52) | 2 | (0-5) | 2 | (1-16) | **2** | **(0-10)** | **7** | **(1-35)+** |
| *Renal replacement therapy (%)* | **18** | **(27)** | **12** | **(52)*** | **0** | **(0)** | **4** | **(36)#** | **22** | **(28)** | **27** | **(48)+** |
| *Length of stay (days)* |  |  |  |  |  |  |  |  |  |  |  |  |
| *In ICU* | **5** | **(2-12)** | **2** | **(1-5)*** | 10 | (1-19) | 1 | (1-10) | **9** | **(3-16)** | **3** | **(1-10)+** |
| *In hospital* | **12** | **(8-28)** | **2** | **(1-5)*** | **21** | **(9-41)** | **1** | **(1-11)#** | **23** | **(12-35)** | **3** | **(1-10)+** |
| *30-day mortality (%)* | **0** | **(0)** | **23** | **(100)*** | **0** | **(0)** | **11** | **(100)#** | **0** | **(0)** | **56** | **(100)+** |
| *Packed red blood cells (n)* | 2 | (0-4) | 3 | (0-9) | 2 | (0-5) | 2 | (1-5) | 2 | (0-6) | 2 | (0-4) |
| *Platelet concentrate (n)* | 2 | (0-4) | 1 | (0-4) | 0 | (0-2) | 0 | (0-1) | **0** | **(0-2)** | **0** | **(0-2)+** |
| *Fresh frozen plasma (n)* | 0 | (0-4) | 0 | (0-0) | 0 | (0-0) | 0 | (0-4) | 0 | (0-0) | 0 | (0-0) |
| *Prothrombin complex concentrate (IU)* | 1500 | (0-3000) | 0 | (0-3000) | 1250 | (0-2000) | 1000 | (0-2000) | 0 | (0-2000) | 0 | (0-2000) |
| *Fibrinogen (g)* | 0 | (0-2) | 0 | (0-0) | 0 | 0(-2) | 0 | (0-0) | 0 | (0-2) | 0 | (0-2) |
| *Recombinant Factor VIIa (IU)* | 0 | (0-0) | 0 | (0-0) | 0 | (0-0) | 0 | (0-0) | 0 | (0-0) | 0 | (0-0) |
| *Antithrombin III (IU)* | 0 | (0-0) | 0 | (0-0) | 0 | (0-0) | 0 | (0-0) | 0 | (0-0) | 0 | (0-0) |
| *Factor XIII (IU)* | 0 | (0-0) | 0 | (0-0) | 0 | (0-0) | 0 | (0-0) | 0 | (0-0) | 0 | (0-0) |
| *Adverse events* |  |  |  |  |  |  |  |  |  |  |  |  |
| *Pneumonia (%)* | 12 | (18) | 3 | (13) | 1 | (6) | 1 | (9) | **9** | **(11)** | **0** | **(0)+** |
| *Pulmonary embolism (%)* | 1 | (1) | 0 | (0) | 0 | (0) | 1 | (9) | 1 | (1) | 1 | (2) |
| *Acute myocardial infarction (%)* | 0 | (0) | 2 | (9) | 0 | (0) | 0 | (0) | 1 | (1) | 0 | (0) |
| *Embolic apoplexy (%)* | 1 | (1) | 2 | (9) | 0 | (0) | 1 | (9) | 0 | (0) | 1 | (2) |
| *Peripheral arterial embolism and thrombosis (%)* | 3 | (4) | 1 | (4) | 0 | (0) | 0 | (0) | 1 | (1) | 1 | (2) |
| *Gastrointestinal ischemia (%)* | **2** | **(3)** | **6** | **(26)*** | 0 | (0) | 0 | (0) | 0 | (0) | 1 | (2) |
| *Gastrointestinal bleeding (%)* | 0 | (0) | 0 | (0) | 0 | (0) | 0 | (0) | 2 | (3) | 1 | (2) |
| *Rotational thromboelastometry* |  |  |  |  |  |  |  |  |  |  |  |  |
| *CT EXTEM* | **130** | **(117-157)** | **171** | **(132-304)*** | **109** | **(102-124)** | **130** | **(119-195)#** | 125 | (107-187) | 136 | (111-181) |
| *CT INTEM* | 330 | (239-433) | 406 | (326-561) | 219 | (184-275) | 249 | (200-339) | **247** | **(194-312)** | **328** | **(243-456)+** |
| *A10 EXTEM* | **52** | **(40-61)** | **44** | **(29-55)*** | 45 | (34-61) | 49 | (30-61) | **50** | **(33-60)** | **39** | **(27-55)+** |
| *A10 FIBTEM* | 13 | (8-18) | 11 | (8-16) | 13 | (4-17) | 7 | (7-27) | **13** | **(5-22)** | **8** | **(4-17)+** |
| *CT APTEM* | **128** | **(115-154)** | **174** | **(124-324)*** | **106** | **(89-116)** | **156** | **(116-168)#** | 127 | (98-164) | 132 | (104-174) |

Appendix 2: Survivor versus non-survivor. Continuous variables are expressed as median and interquartile range and were compared with Mann Whitney U test. Categorical variables are presented as numbers (column percentages) and were compared with chi^2^ tests. ICU (Intensive care unit). Packed red blood cells (450mL), Platelet concentrate (270mL), Fresh frozen plasma (300mL). APTEM: extrinsic pathway with inhibition of fibrinolysis. EXTEM: extrinsic pathway. FIBTEM: fibrin contribution to clot firmness. INTEM: intrinsic pathway without heparin neutralization. The leading department is selected in all 3 groups. Groupe 1: Cardiothoracic surgery with CPB includes patients undergoing surgery with cardiopulmonary bypass (CPB). Groupe 2: Trauma includes traumatically injured patients and with 115 patients suffering from multiple injuries that involve multiple organs. Groupe 3: Medical bleeding includes all patients with surgery without cardiopulmonary bypass (general surgery (without liver), liver surgery, orthopedic surgery, pediatric, eye, thoracic surgery without CPB, vascular surgery without CPB, gynecology, urology, neurosurgery, ear, nose, throat, and maxillofacial surgery) and 79 patients from internal medicine. * p<0.05 versus CT_EXTEM_ < 110s. # p<0.05 versus CT_EXTEM_ < 98s. + p<0.05 versus CT_EXTEM_ < 99s.
